# Effects of Lactoferrin Supplemented with Fermented Milk on Obesity-Associated Pancreatic Damage in Rats

**DOI:** 10.3390/life12122019

**Published:** 2022-12-03

**Authors:** Mona A. Hassan, Tarek Gamal Abedelmaksoud, Ahmed A. Abd El-Maksoud

**Affiliations:** 1 Food Evaluation and Food Science Department, National Organization for Drug Control and Research, Giza 12553, Egypt; 2 Food Science Department, Faculty of Agriculture, Cairo University, Giza 12613, Egypt; 3 Dairy Science Department, Faculty of Agriculture, Cairo University, Giza 12613, Egypt

**Keywords:** natural protective, stirred yogurt, high-fat diet, non-alcoholic fatty pancreas

## Abstract

Non-alcoholic fatty pancreas disease is a newly emerging disease that represents an important risk factor for the development of pancreatic cancer. Obesity is a risk factor for pancreatic diseases, including pancreatitis and pancreatic cancer. On the other hand, the development of healthy aspects-based food products is a recent trend. Lactoferrin is a component of the body’s immune system, which interacts with DNA, RNA, polysaccharides, and heparin, and it has many biological functions and many important immunomodulatory properties. Thus, this study aims to investigate the enhancement effect of supplementation of lactoferrin with stirred yogurt on weight gain, lipid profile, glucose level, and pancreatic enzymes in animals fed a high-fat diet (HFD). Forty-eight female albino rats were divided into 6 groups treated orally for 45 days as follows: negative control (basal diet), positive control (add 1% cholesterol), stirred yogurt (SY), Lactoferrin LF (100 mg/kg bw), supplementation of lactoferrin with stirred yogurt SY–LF at two concentrations LF1 (50 mg/kg bw) and LF2 (100 mg/kg bw). Blood and pancreas samples were collected for different analyses. Animals fed with a HFD showed a significant increase in body weight, total cholesterol, triglyceride, low-density lipoprotein (LDL), glucose level, amylase, and Lipase enzymes (44.72%, 151.33 mg/dL, 142.67 mg/dL, 85.37 mg/dL, 141.33 mg/dL, 39.33 U/mL, 23.43 U/mL). Moreover, it observed a significant decrease in high-density lipoprotein (HDL, 37.33 mg/dL); meanwhile, SY fortified with lactoferrin was useful in losing weight gain and improving lipid profile, pancreas function, and histological change in the pancreas. The supplementation of lactoferrin at 100 mg/Kg bw with LB. Acidophilus as a probiotic was more effective for pancreas functions. This application is a natural protective alternative to manufactured medicines for children and the elderly as a natural product.

## 1. Introduction

Recently, several studies provided strong evidence that trypsinogen activation is likely an important first step in the inflammatory cascade underlying pancreatitis and that persistent pancreatitis depends on cytokine activation associated with damage-associated molecular patterns by an innate factor revealing chronic inflammation and fibrosis. It depends on the generation of IL-33 by infected acinar cells and its final induction of IL-13-producing T cells. In addition, recent studies have revealed that pancreatitis is a unique and completely different form of inflammation and that it is subject to newer and more innovative treatment. Inflammation and autophagy processes are disorganized (underactive or overactive), and they also lead to pathological effects such as oxidative stress, cell death, and metabolic impairment [1]. In addition, obesity is a risk factor that promotes inflammation and development the pancreatic diseases [2]. The immune system plays a complex role in the progression of pancreatic cancer and the immune response is involved in pancreatitis and pancreatic cancer, including the role of specific cytokines and implications for disease outcome. Acute pancreatitis is characterized by an innate immune response, while chronic pancreatitis causes an immune response involving both innate and adaptive immune cells. Pancreatic adenocarcinoma is characterized by a dysfunction of the immune system caused by some types of immunosuppressive cells, defective or absent inflammatory cells, and tumor-promoting immune cells. Recent studies have revealed that immune cells interact with cancer stem cells and tumor stromal cells, as well as there being an effect of these interactions on the development of pancreatic ductal adenocarcinoma progression. Acute pancreatitis (AP) is a major cause of increased morbidity and mortality [3]. The pathophysiology (AP) is rather complex, which limits treatment options significantly. Several studies have revealed that infiltrating immune cells play an important role in the pathogenesis of AP and in determining disease severity. Therefore, it is expected that immunotherapy targeting immune cells and associated inflammatory mediators will be a new treatment modality for AP that may improve patients’ prognosis [4]. Pancreatitis is a common disorder with significant morbidity and mortality, and there is no specific or effective treatment, as it is clear from studies in genetic and experimental mouse models that it develops irregular autophagy that leads to enhanced inflammatory response in the pancreas [5]. Obesity is a risk factor for pancreatic disease, including pancreatitis and pancreatic cancer. Severe acute pancreatitis is more common in obese patients and being overweight or obese is associated with an increased risk of pancreatic cancer and a negative risk factor for increased mortality in pancreatic cancer [6]. Pancreatic steatosis is associated with several congenital and acquired factors, including cystic fibrosis, age, viral infections, medications, and excessive alcohol consumption [7], but the association with metabolic factors (obesity and T2DM) has led to renewed interest and research. Non-alcoholic fatty liver disease (NAFPD) may increase the risk and severity of pancreatitis, beta-cell 13-cell dysfunction, T2DM development, and pancreatic cancer [8]. Pancreatic adenocarcinoma (PDAC) is one of the deadliest types of cancer and is the third most common cause of cancer death in the United States of America. One of the causes of PDAC is the deposition of excess fat within the pancreas i.e., the fatty pancreas that particularly affects the tumor. Patients with acute pancreatitis with diabetes have been noted to have elevated fat levels in the pancreas. It was found that through obesity there is a strong association between pancreatic fat and fatty liver [9]. Obesity and pancreatic cancer risk are linked by numerous hormonal and inflammatory effects of adipose tissue, reduced physical activity, and obesity-induced hypoxia, which leads to increases in vascular endothelial growth factor [10].

Lactoferrin (LF) is a protein found in milk and mammalian secretion fluids; lactoferrin is an iron-binding glycoprotein that aids in the effect of precursor cells to develop helper T-cell polarization that has a role in maintaining immune homeostasis, especially as a part of the immune system’s response to infection, trauma, and injury [11]. LF is also well-known for its various biological activities, which include antibacterial action, and it has immunological activity. LF interacts with DNA, RNA, polysaccharides, and heparin, and has many biological functions in complexes with these bonds. LF is considered a supplement that reduces the risk of respiratory infections from a recent meta-analysis of randomized controlled trials [12]. Moreover, there is evidence that it binds to the receptors used by coronaviruses and thus prevents their entry, and it has a preventive and therapeutic value during the current COVID-19 pandemic [13]. Preliminary studies revealed the beneficial effect on lipid metabolism and obesity in a small number of volunteers and the trials were conducted in Japan in 2003. Positive results were also supported by laboratory tests recording decreases in body weight, visceral fat tissue, waist measurement, plasma concentrations, liver fatty acids, cholesterol, and triglycerides. In addition, LF improves high HDL cholesterol, which has antiatherosclerosis properties, inhibits oxidized LDL cholesterol, and prevents the production of foamy cells. LF also increases the susceptibility of cells to the effects of insulin, including decreased response to insulin during inflammation, and it improved hepatic insulin resistance and pancreatic dysfunction [14,15]. From the previous studies, there is no study available regarding the effect of lactoferrin supplemented with stirred yogurt on obesity-associated pancreatic damage. Therefore, this study aims to investigate the enhancement effect of supplementation of lactoferrin with stirred yogurt on weight gain, lipid profile, glucose level, and pancreas enzymes in animals fed a high-fat diet (HFD).

## 2. Materials and Methods

### 2.1. Materials

Buffalo raw milk was obtained from the herd of the Agriculture Research Station, Cairo University (Egypt). Lyophilized starter culture contains equal mixtures of *Streptococcus thermophilus* and *Lactobacillus delbrueckii* subspp. *bulguricus* YoFlex^®^ and LB. Acidophilus was obtained from ChrHansen, Denmark. Lactoferrin was obtained from Sigma-Aldrich Co., St. Louis, MO, USA. Aspartate Transaminase and Alanine Aminotransferase kits were purchased from Randox, Antrim (UK Co.). Cholesterol 1% purchased from Sigma Chemical Co. (St. Luis, MO, USA). Moreover, the cholesterol (CHO), triglycerides (Tri. G), high-density lipoprotein (HDL), and low-density lipoprotein (LDL) kits were purchased from FAR Diagnostics Co. (Via Fermi, Italy). Glucose, Lipase, and Amylase kits Bio Med-Diagnostics were purchased from EGY-CHEM for lab technology.

### 2.2. Methods

#### 2.2.1. Preparation of Stirred Yogurt (SY)

Buffalo’s skimmed milk was used for stirred yogurt manufacturing. Skimmed milk was pasteurized at 85 °C for 5 min after cooling, and pure Lactoferrin (LF) was mixed with skimmed milk sample at a concentration of 500 mg LF/1L skimmed milk. Two samples of stirred yogurt were prepared (stirred yogurt alone SY, stirred yogurt fortified with lactoferrin, SY-LF). Direct vat set yogurt culture containing *Lactobacillus delbrueckii* subsp. *bulgaricus* and *Streptococcus thermophiles* with LB. Acidophilus (LA, probiotic strain) was enriched and propagated in sterilized skimmed milk. The propagated culture was inoculated to the skimmed milk in 1.5%, incubated at 42 °C until completely coagulated and the pH reached 4.6. The samples were mixed with an electric stirrer and then packed in 100 mL sterilized cups and stored in a refrigerator at 4 °C for further analyses.

#### 2.2.2. Chemical Analysis of Stirred Yogurt and Stirred Yogurt Fortified with Lactoferrin

Total solids, ash, pH values, and protein contents were determined according to the A.O.A.C. [16], and total carbohydrate was determined according to DuBois et al. [17].

#### 2.2.3. Determination of Lactoferrin in Stirred Yogurt and Stirred Yogurt Fortified with Lactoferrin

Lactoferrin levels were determined by using a commercial lactoferrin ELISA kit through a sandwich technique according to Cheng et al. [18].

#### 2.2.4. Determination of Glucose Level and Pancreas Enzymes

Lipase and amylase serum levels were measured by enzymatic colorimetric test by following the method of Lott et al. [19] and Kurahashi and Inomata [20], respectively. Serum glucose was assayed calorimetrically according to the method of Trinder [21].

#### 2.2.5. Experimental Animals

Adult female albino Wistar rats were obtained from the National Organization for Drug Control and Research (NODCR) and were weighed (130 ± 10 g) to use them for the experiments. Rats were housed in hanging wire mesh cages in a temperature (23 ± 2 °C) and humidity (50 ± 5%) controlled room with a 12:12-h light-dark cycle. Animals were fed on a standard lab diet purchased from Meladco Feed Co, Aubor City, FL, USA Cairo throughout the experimental period. The rats were allowed to adapt to our laboratory environment for 1 week before beginning the experiment.

These animals received humane care in compliance with the guidelines of the Animal Care Using Committee of the National Research Centre and the National Institutes of Health (Committee of general division for Biological Control and Research, No 253, Date 29 December 2021).

#### 2.2.6. Experimental Design

Forty-eight rats were randomly allocated into six groups. Orally, all treated animals were fed a high-fat diet (HFD) for 45 days. The first group received a standard lab diet as normal (control group), the second group of animals were treated with cholesterol (1%) according to Chowdhury and Forsmark [22], which this is the most often used obesity model worldwide; the third group of animals was treated with stirred yogurt (0.5 mL /kg bw); the fourth group of animals was treated with lactoferrin alone 0.5 mL (100 mg/kg bw; the fifth group of animals was treated with 0.5 mL stirred yogurt fortified with lactoferrin LF1 (50 mg/kg bw); and the sixth group of animals was treated with 0.5 mL stirred yogurt fortified with lactoferrin at 100 mg/kg bw concentrate (LF2). The initial and final body weights of the rats in the different groups are presented. All animals fasted for 12 h, and then blood samples were collected from the retro-orbital venous plexus under diethyl ether anesthesia for determination of lipid profile, glucose levels, and pancreas enzymes. Animals were sacrificed by cervical dislocation and samples of the pancreas were collected. These samples were fixed in 10% neutral formalin and paraffin-embedded. Sections (5 μm thickness) were stained with hematoxylin and eosin (Hx and E) for the histological examination of pancreas according to Helal, Sharaf, and Mattar [23].

#### 2.2.7. Statistical Analysis

All obtained data were expressed as mean ± standard deviation of triplicate independent experiments. Statistics were performed using SPSS system 11.5 (SPSS, Inc. © Chicago, IL, USA). Means were compared using Duncan’s multiple range test by one-way analysis of variance (ANOVA), which was carried out to determine the significant differences among the samples; differences were considered significant at *p* < 0.05 [24].

## 3. Results

### 3.1. Chemical Composition and PHYSICAL Properties of Stirred Yogurt (SY) and Stirred Yogurt Fortified with Lactoferrin (SY-LF)

Total solids, protein, fat content, carbohydrate, ash, and pH values were presented in Table 1. The chemical composition was within the normal range in the SY-LF. Additionally, the total protein and ash were significantly increased in the SY–LF samples compared to SY alone.

### 3.2. The Effect of Fermentation on the Concentration of Lactoferrin in SY and SY-LF

**Figure 1:**
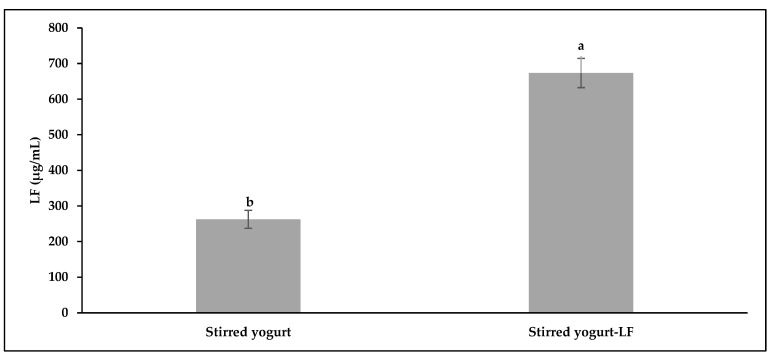
The concentration of lactoferrin in the SY and SY-LF; the results represent the mean ± SD (n = 3). The different letters above the bars indicate a significant difference (*p* < 0.05).

The effect of fermentation on the concentration of lactoferrin in the studied samples is presented in Figure 1. From the data, it can be noticed that the efficiency of lactoferrin in the SY–LF sample was higher than 98%, which indicates that the fermentation process did not affect the lactoferrin in stirred yogurt.

### 3.3. The Effect of LF Supplemented with Skim Milk on the Viability of Probiotic Starter after Fermentation

**Figure 2:**
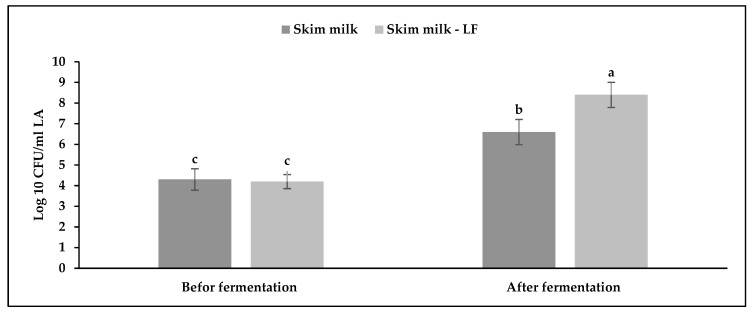
The viable counts of LB. Acidophilus (Log cfu/mL) in skimmed milk before and after fermentation. The results represent the mean ± SD (n = 3). The different letters above the bars indicate a significant difference (*p* < 0.05).

The effect of LF addition on the viability of LB. Acidophilus (LA) starter in the skimmed milk samples after fermentation was evaluated as shown in Figure 2. The results indicated that the mean values of viable counts of LA were increased after the fermentation period conducted at 42 °C for 3.5 h in addition to the yogurt starter (*L. Bulgaricus* and *S. thermophilus*). The levels of the viable counts of LA in the SY–LF sample were significantly increased in comparison with SY alone and these results indicated that the existence of LF on fermented milk was improved the growth of the LA starter due to the prebiotic effects of LF on enrichment fermented culture (Figure 2).

### 3.4. The Effect of Lactoferrin Supplemented with Stirred Yogurt on the Body Weight

The initial and final body weights of the animals in the different groups are presented in Table 2. In the current study, animals fed on a HFD showed a significant increase in body weight gain in comparison with control animals and those treated with SY-LF1 and SY-LF2. The weight gain in the animals treated with SY-LF1 and SY-LF2 for (6 weeks) was shown to significantly decrease in weight gain compared to the HFD-fed animals.

### 3.5. The Effect of Lactoferrin Supplemented with SY on Serum Lipid Levels

The lipid profile of the animals treated with SY–LF and a HFD is presented in Table 3. In the current study, the animals that were fed a HFD saw a significant increase in total cholesterol, triglyceride, and low-density lipoprotein, while there was a decrease in the high-density lipoprotein compared to the control and the groups treated with SY-LF1 and SY- LF2. The supplementation with LF1 and LF2 in the two concentrations with stirred yogurt significantly improved the lipid profile; meanwhile, treatment with SY-LF2 was more effective than other treatments in the lipid profile.

### 3.6. The Effect of Lactoferrin Supplemented with SY on Glucose and Pancreas Enzymes

As shown in Table 4, the level of serum glucose, amylase, and lipase in animals that were fed on a HFD significantly increased compared to the control animals and those treated with SY-LF1 and SY-LF2. The supplementation of stirred yogurt with LF1 and LF2 decreased the glucose, amylase, and lipase compared to the HFD-fed animals group. Additionally, the obtained results of the groups treated with SY-LF1 and SY-LF2 were similar to the control animals’ group on the levels of glucose and pancreatic enzymes.

### 3.7. Histological Examination

**Figure 3:**
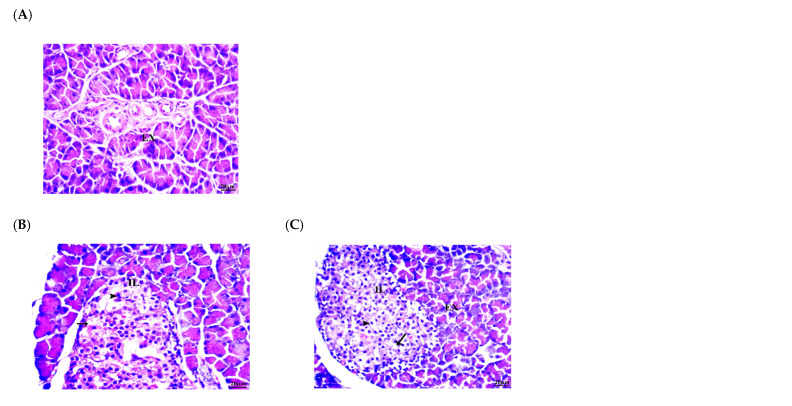

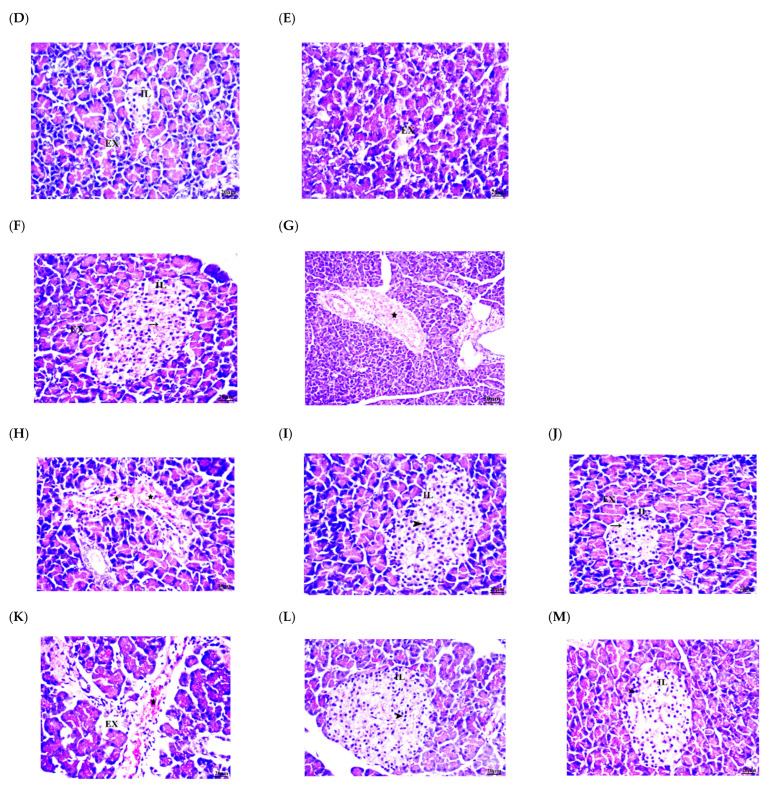
Photomicrograph of pancreas sections of treated groups. The control group (**A**); group fed high-fat diet figs (**B**,**C**); group treated with stirred yogurt alone figs (**D**,**E**); groups received lactoferrin alone (100 mg/Kg bw) (**F**,**G**); group treated with stirred yogurt fortified with lactoferrin (50 mg/Kg bw) (**H**–**J**) and group treated with stirred yogurt fortified with lactoferrin (100 mg/Kg bw) (**K**–**M**); EX: exocrine pancreas (acinar cells); IL: islets of Langerhans; Arrow: vacuolation; Arrow head: beta cells; Star: congestion.

The histological study of pancreas sections is presented in Table 5 and Figure 3. The results showed the micrograph of the pancreas samples stained with hematoxylin and eosin. In the current study, the control group is presented in Figure 3A and the pancreatic architecture in the control group is normal. Additionally, the exocrine component of the pancreas is organized into tiny lobules and densely packed by acinar cells. The group fed a HFD (Figure 3B,C) showed that the cholesterol group has a distorted overall architecture. The majority of exocrine acini showed acinar injury in the form of cell atrophy. Islets of Langerhans shrink as a result of degeneration, vacuolation, and necrosis of the components. The group treated with stirred yogurt alone is shown in Figure 3D,E, and there was no improvement in pancreatic histology, moderate acinar cells degeneration, and islets of Langerhans. Meanwhile, the groups that received lactoferrin alone at 100 mg/Kg bw are shown in Figure 3F,G, and the group treated with stirred yogurt fortified with lactoferrin at 50 mg/Kg bw is presented in Figure 3H–J. Moreover, the group treated with stirred yogurt fortified with lactoferrin at 100 mg/Kg bw, shown in Figure 3K–M, showed that it was able to return to a state that was close to normal. The islets of Langerhans were typical in size, although there was considerable degradation of the β cell in the middle. Little vacuolation was seen within islets of Langerhans and minor congestion was observed in all treated groups.

## 4. Discussion

Acute pancreatitis is one of the most common diseases in obese patients [6]. As shown in the present study, animals that were fed on a HFD recorded a significant increase in body weight and lipid levels including total cholesterol, triglyceride, and LDL, and a significant decrease in HDL. Moreover, there was a significant increase in serum glucose concentration in the animals that were fed a HFD. These results are supported by previous studies, which reported that rats fed a HFD consumed more calories than the basal diet group or rats fed a yogurt supplement that reduced calorie intake [25]. This is due to the different metabolic activities of some fatty acids of a HFD, which can alter both fat oxidation and deposition rates, resulting in changes in body weight and fat composition [26].

Rats that were fed a HFD showed an increase in glucose levels and plasma lipid levels, which included cholesterol and triglyceride levels, and saturated fat was responsible for the increase in glucose and lipid levels [25,27]. In addition, a prior study found that a chemical examination of pancreatic fat revealed an increase in poly-unsaturated fatty acids in pancreatic triglycerides in persons with higher pancreatic fat levels compared to those with lower levels [28]. In the current study, supplemented lactoferrin with stirred yogurt significantly decreased weight gain, serum glucose concentration, and lipid levels by restoring the cellular antioxidant in the obese animals; these results agree with the studies suggesting that Lactoferrin produced sustained weight and fat loss. Interestingly, lactoferrin improves post-meal glucose clearance in part through the regulation of the liver and muscle glycogen genes as well as its role in improving glycemic control in diet-induced rats [29]. Taking lactoferrin supplements for 8 weeks has been shown to reduce visceral obesity in overweight subjects without a change in caloric intake [30]. As shown in the current study, the levels of lipase and amylase serum were significantly increased in animals that consumed a high-fat diet. These results are supported by previous studies, which reported that two prospective cohort studies demonstrated that obesity significantly increased the risk of pancreatic cancer, and that physical activity reduces the risk of pancreatic cancer, especially for those who are overweight [31]. Acute pancreatitis (AP) has many diverse etiologies and outcomes. Numerous studies have reported that obese patients, who have increased visceral fat stores including pancreatic fat, are at risk of developing severe acute pancreatitis SAP [32,33]. The study reported that the cleaning fluid for pancreatic necrosis contains a high concentration of unsaturated fatty acids [34]. Thus, our findings suggested that lactoferrin at two doses (50, 100 mg/kg bw) supplemented with stirred yogurt normalized the pancreatic enzymes and reduced the complication of pancreas damage. These are consistent with previous studies, showing that the results explain the detection and immunological effect of lactoferrin in pancreatic juice for patients, especially in cases of chronic pancreatitis [35].

## 5. Conclusions

During this study, the effect of lactoferrin supplementation with stirred yogurt on weight gain, lipid profile, glucose level, and pancreas enzymes in animals fed on high-fat diet (HFD) was investigated. Animals fed with a HFD showed a significant increase in body weight, total cholesterol, triglyceride, low-density lipoprotein LDL, glucose level, Lipase, and amylase enzymes, and also significantly decreased high-density lipoprotein HDL. Therefore, it can be concluded that supplementation of lactoferrin with stirred yogurt has protective effects against obesity, improving lipid profile, and pancreas function, and that it may be a valuable prospect as a novel ingredient for the development of functional fermented milk products. This application is an alternative to manufactured medicines for children and the elderly as a natural product.

## Figures and Tables

**Table 1:** Chemical composition and physical properties of SY and SY-LF.

Parameter	Total Solid	Protein	Fat	Carbohydrate	Ash	pH
SY	13.41 ± 0.21 ^a^	4.23 ± 0.18 ^b^	0.21± 0.02 ^a^	6.7 ± 0.28 ^a^	0.86 ± 0.02 ^b^	4.52 ± 0.21 ^a^
SY + LF	13.34 ± 0.16 ^a^	4.83 ± 0.12 ^a^	0.24 ± 0.02 ^a^	6.5 ± 0.18 ^a^	0.97 ± 0.03 ^a^	4.55 ± 0.10 ^a^

The results represent the mean ± SD (n = 3). Different superscript letters in the same column indicate a significant difference (*p* < 0.05). SY: stirred yogurt; LF: lactoferrin.

**Table 2:** The effect of lactoferrin supplemented with SY on body weight.

Weight (g)	−Control	+Control (HFD)	SY	LF	SY-LF1	SY-LF2
Initial weight	139.67 ± 2.06 ^a^	129.87 ± 0.65 ^b^	119.22 ± 0.04 ^c^	119.08 ± 0.26 ^c^	119.96 ± 0.22 ^c^	129.9 ± 0.17 ^b^
Final weight	167 ± 4.02 ^b^	188 ± 2.01 ^a^	159.67 ± 5.51 ^bc^	162 ± 2.14 ^b^	153 ± 3.57 ^c^	161.33 ± 1.16 ^b^
Weight gain %	19.51 ± 2.05 ^e^	44.72 ± 1.95 ^a^	33.7 ± 4.45 ^bc^	36.03 ± 2.05 ^b^	27.54 ± 4.05 ^cd^	24.19 ± 1.24 ^d^

The results represent the mean ± SD (n = 3), the different letters above the bars indicate a significant difference (*p* < 0.05). SY: stirred yogurt; LF: lactoferrin.

**Table 3:** The effect of lactoferrin supplemented with SY on serum Lipid Profile.

Parameter	−Control	+Control (HFD)	SY	LF	SY-LF1	SY-LF2
Cholesterol (mg/dL)	95.67 ± 0.58 ^d^	151.33 ± 1.53 ^a^	110.67 ± 1.15 ^b^	109.00 ± 1.90 ^b^	102.67 ± 1.53 ^c^	102.00 ± 1.88 ^c^
Triglyceride (mg/dL)	89 ± 1 ^c^	142.67 ± 1.53 ^a^	93.33 ± 0.58 ^c^	101.33 ± 2.52 ^b^	91 ± 2.00 ^cd^	91.33 ± 1.15 ^c^
HDL (mg/dL)	44.33 ± 0.58 ^a^	37.33 ± 1.15 ^c^	41 ± 1 ^b^	42.67 ± 2.52 ^ab^	43 ± 1 ^ab^	43.67 ± 0.58 ^a^
LDL (mg/dL)	34.23 ± 0.15 ^e^	85.37 ± 0.91 ^a^	47.67 ± 0.46 ^b^	48.10 ± 0.87 ^b^	41.47 ± 0.31 ^c^	39.73 ± 0.50 ^d^

The results represent the mean ± SD (n = 3), the different letters above the bars indicate a significant difference (*p* < 0.05). SY: stirred yogurt; LF: lactoferrin.

**Table 4:** The effect of lactoferrin supplemented with SY on glucose levels and pancreatic enzymes.

Parameter	−Control	+Control (HFD)	SY	LF	SY-LF1	SY-LF2
Glucose (mg/dL)	80.00 ± 1.00 ^c^	141.33 ± 2.08 ^a^	82.33 ± 1.53 ^bc^	85.67 ± 3.06 ^b^	81.67 ± 2.08 ^bc^	80.00 ± 2.00 ^c^
Amylase (U/mL)	27.20 ± 0.60 ^c^	39.33 ± 0.68 ^a^	29.53 ± 0.52 ^b^	26.93 ± 1.31 ^c^	27.60 ± 1.11 ^c^	26.90 ± 1.50 ^c^
Lipase (U/mL)	10.28 ± 0.12 ^c^	23.43 ± 2.11 ^a^	10.73 ± 0.15 ^b^	10.24 ± 0.07 ^c^	10.36 ± 0.09 ^c^	10.27 ± 0.04 ^c^

The results represent the mean ± SD (n = 3), the different letters above the bars indicate a significant difference (*p* < 0.05). SY: stirred yogurt; LF: lactoferrin.

**Table 5:** Effect of lactoferrin supplementation with SY on the pancreas.

Congestion	Degenerated	Vacuolation	Groups
Control	−	−	−
HFD	+++	++	++
SY	+++	++	++
LF	++	+	+
SY-LF2	+	+	+
SY-LF1	+	+	+

Note: HFD: high fat diet; SY: stirred yogurt; LF: lactoferrin; The scores were estimated as follows: absent: −, mild: +, moderate: ++, severe: +++.

## Data Availability

Not applicable.
